# Poly[[tetra­aqua­(μ_4_-imidazole-4,5-dicarboxyl­ato)(μ_3_-imidazole-4,5-dicarboxyl­ato)-μ_3_-sulfato-μ_2_-sulfato-cobalt(II)digadolinium(III)] monohydrate]

**DOI:** 10.1107/S160053681104726X

**Published:** 2011-11-12

**Authors:** Li-Cai Zhu

**Affiliations:** a School of Chemistry and Environment, South China Normal University, Guangzhou 510631, People’s Republic of China

## Abstract

The asymmetric unit of the title compound, {[CoGd_2_(C_5_H_2_N_2_O_4_)_2_(SO_4_)_2_(H_2_O)_4_]·H_2_O}_*n*_, contains one Co^II^ ion, two Gd^III^ ions, two imidazole-4,5-dicarboxyl­ate ligands, two SO_4_^2−^ anions, four coordinated water mol­ecules and one uncoordinated water mol­ecule. The Co^II^ ion is six-coordinated by two O atoms from two coordinated water mol­ecules, as well as two O atoms and two N atoms from two imidazole-4,5-dicarboxyl­ate ligands, giving a slightly distorted octa­hedral geometry. Both Gd^III^ ions are eight-coordinated in a distorted bicapped trigonal–prismatic geometry. One Gd^III^ ion is coordinated by four O atoms from two imidazole-4,5-dicarboxyl­ate ligands, three O atoms from three SO_4_^2−^ anions and a water O atom; the other Gd^III^ ion is bonded to five O atoms from three imidazole-4,5-dicarboxyl­ate ligands, two O atoms from two SO_4_^2−^ anions as well as a water O atom. These metal coordination units are connected by bridging imidazole-4,5-dicarboxyl­ate and sulfate ligands, generating a heterometallic layer parallel to the *ac* plane. The layers are stacked along the *b* axis *via* N—H⋯O, O—H⋯O, and C—H⋯O hydrogen-bonding inter­actions, generating a three-dimensional framework.

## Related literature

For applications of lanthanide–transition metal heterometallic complexes with bridging multifunctional organic ligands, see: Cheng *et al.* (2006 ▶); Kuang *et al.* (2007 ▶); Sun *et al.* (2006 ▶); Zhu *et al.* (2010 ▶).
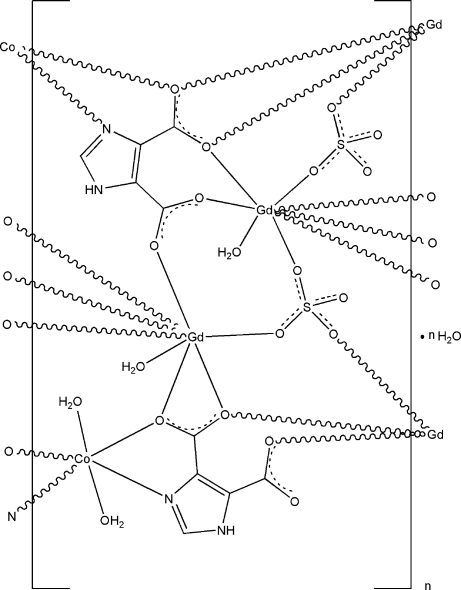


## Experimental

### 

#### Crystal data

[CoGd_2_(C_5_H_2_N_2_O_4_)_2_(SO_4_)_2_(H_2_O)_4_]·H_2_O
*M* *_r_* = 963.82
Triclinic, 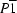

*a* = 9.0916 (5) Å
*b* = 10.7714 (6) Å
*c* = 12.9736 (7) Å
α = 93.119 (1)°
β = 96.416 (1)°
γ = 108.840 (1)°
*V* = 1189.35 (11) Å^3^
*Z* = 2
Mo *K*α radiation
μ = 6.48 mm^−1^
*T* = 296 K
0.20 × 0.18 × 0.15 mm

#### Data collection

Bruker APEXII area-detector diffractometer
Absorption correction: multi-scan (*SADABS*; Sheldrick, 1996 ▶) *T*_min_ = 0.284, *T*_max_ = 0.378
6174 measured reflections
4208 independent reflections
3790 reflections with *I* > 2σ(*I*)
*R*_int_ = 0.016

#### Refinement

*R*[*F* ^2^ > 2σ(*F* ^2^)] = 0.022
*wR*(*F* ^2^) = 0.054
*S* = 1.02
4208 reflections
397 parameters
17 restraints
H atoms treated by a mixture of independent and constrained refinement
Δρ_max_ = 0.79 e Å^−3^
Δρ_min_ = −0.81 e Å^−3^

### 

Data collection: *APEX2* (Bruker, 2004 ▶); cell refinement: *SAINT* (Bruker, 2004 ▶); data reduction: *SAINT*; program(s) used to solve structure: *SHELXS97* (Sheldrick, 2008 ▶); program(s) used to refine structure: *SHELXL97* (Sheldrick, 2008 ▶); molecular graphics: *XP* in *SHELXTL* (Sheldrick, 2008 ▶); software used to prepare material for publication: *SHELXL97*.

## Supplementary Material

Crystal structure: contains datablock(s) I, global. DOI: 10.1107/S160053681104726X/hp2018sup1.cif

Structure factors: contains datablock(s) I. DOI: 10.1107/S160053681104726X/hp2018Isup2.hkl

Additional supplementary materials:  crystallographic information; 3D view; checkCIF report

## Figures and Tables

**Table 1:** Hydrogen-bond geometry (Å, °)

*D*—H⋯*A*	*D*—H	H⋯*A*	*D*⋯*A*	*D*—H⋯*A*
N1—H1⋯O1^i^	0.87 (4)	1.96 (4)	2.820 (5)	172 (5)
O1*W*—H1*W*⋯O5*W*^ii^	0.81 (4)	1.95 (3)	2.745 (5)	169 (6)
N3—H2⋯O14^iii^	0.87 (3)	1.93 (3)	2.787 (4)	169 (4)
O2*W*—H3*W*⋯O1^i^	0.80 (3)	2.09 (4)	2.878 (5)	171 (5)
O2*W*—H4*W*⋯O14^i^	0.81 (4)	2.04 (4)	2.842 (5)	172 (5)
O2*W*—H4*W*⋯O15^i^	0.81 (4)	2.52 (4)	3.035 (5)	123 (4)
O3*W*—H5*W*⋯O12^iv^	0.82 (3)	1.95 (4)	2.734 (4)	162 (5)
O3*W*—H6*W*⋯O14^v^	0.83 (3)	2.41 (4)	2.919 (4)	120 (3)
O4*W*—H7*W*⋯O3^vi^	0.82 (3)	2.49 (3)	3.306 (6)	174 (6)
O4*W*—H8*W*⋯O5*W*^iii^	0.82 (5)	1.89 (5)	2.700 (6)	175 (6)
O5*W*—H9*W*⋯O12^v^	0.85 (4)	1.99 (4)	2.797 (6)	161 (5)
O5*W*—H10*W*⋯O1^i^	0.85 (5)	1.93 (5)	2.728 (5)	157 (6)
C3—H3⋯O3^vii^	0.93	2.44	3.193 (5)	138

Symmetry codes: (i) 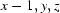; (ii) 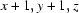; (iii) 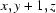; (iv) 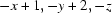; (v) 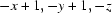; (vi) 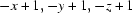; (vii) 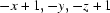.

**Fig. 1.**
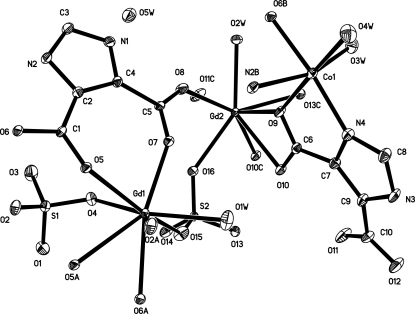
The molecular structure showing the atomic-numbering scheme and displacement ellipsoids drawn at the 30% probability level. Symmetry codes: (A) 2 - x, 1 - y, 1 - z; (B) 1 - x, 1 - y, 1 - z; (C) 1 - x, 1 - y, -z.

**Fig. 2.**
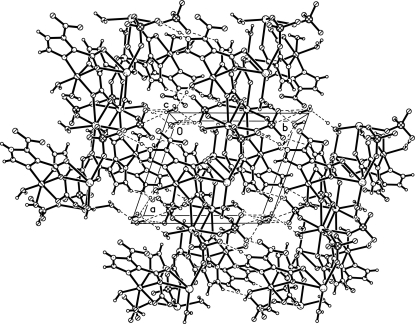
A view of the three-dimensional structure of the title compound, the hydrogen bonding interactions showed as broken lines.

## supplementary crystallographic information

### Comment

In the past few years, lanthanide-transition metal heterometallic complexs with bridging multifunctionnal organic ligands are of increasing interest, not only because of their impressive topological structures, but also due to their versatile applications in ion exchange, magnetism, bimetallic catalysis and luminescent probe(Cheng *et al.*, 2006; Kuang *et al.*, 2007; Sun *et al.*, 2006; Zhu *et al.*, 2010). As an extension of this research, the structure of the title compound, a new heterometallic coordination polymer, (I), has been determined which is presented in this artcle.

The asymmetric unite of the title compound (Fig. 1), contains one Co^II^ ion, two Gd^III^ ions, two imidazole-4, 5-dicarboxylate ligands, two SO_4_^2-^ anions, four coordinated water molecules and one uncoordinated water molecule. The Co^II^ ion is six-coordinated with two O atoms from two coordinated water molecules, two O atoms and two N atoms from two imidazole-4, 5-dicarboxylate ligands, giving a slightly distorted octahedral geometry. Both Gd^III^ ions are eight-coordinated in a bicapped trigonal prismatic coordination geometry. One Gd^III^ ion is coordinated by four O atoms from two imidazole-4,5-dicarboxylate ligands, three O atoms from three SO_4_^2-^ anions and one water molecule; the other Gd^III^ ion is bonded to five O atoms from three imidazole-4, 5-dicarboxylate ligands, two O atoms from two SO_4_^2-^ anions as well as one coordinated water molecule. These metal coordination units are connected by bridging imidazole-4, 5-dicarboxylate and sulfate ligands, generating a two-dimensional heterometallic layer. The two-dimensional layers are stacked along *b* axis *via* N—H···O, O—H···O, and C—H···O hydrogen-bonding interactions to generate the three-dimensional framework(Table 1 and Fig. 2).

### Experimental

A mixture of CoSO_4_.7H_2_O(0.141 g, 0.5 mmol), Gd_2_O_3_(0.09 g, 0.25 mmol), imidazole-4,5-dicarboxylic acid (0.156 g, 1 mmol), and H_2_O(7 ml) was sealed in a 20 ml Teflon-lined reaction vessel at 443 K for 5 days then slowly cooled to room temperature. The product was collected by filtration, washed with water and air-dried. Red block crystals suitable for X-ray analysis were obtained.

### Refinement

H atoms bonded to C atoms were positioned geometrically and refined as riding, with C—H = 0.93 Å and *U*_iso_(H) = 1.2 *U*_eq_(C). H atoms bonded to N atoms and H atoms of water molecules were found from difference Fourier maps and refined isotropically with a restraint of N—H = 0.87 Å, O—H = 0.82 or 0.86 Å and *U*_iso_(H) = 1.5 *U*_eq_(N, O).

### Crystal data

**Table:**

[CoGd_2_(C_5_H_2_N_2_O_4_)_2_(SO_4_)_2_(H_2_O)_4_]·H_2_O	*Z* = 2
*M**_r_* = 963.82	*F*(000) = 914
Triclinic, *P*1	*D*_x_ = 2.691 Mg m^−^^3^
Hall symbol: -P 1	Mo *K*α radiation, λ = 0.71073 Å
*a* = 9.0916 (5) Å	Cell parameters from 4033 reflections
*b* = 10.7714 (6) Å	θ = 2.4–27.9°
*c* = 12.9736 (7) Å	µ = 6.48 mm^−^^1^
α = 93.119 (1)°	*T* = 296 K
β = 96.416 (1)°	Block, red
γ = 108.840 (1)°	0.20 × 0.18 × 0.15 mm
*V* = 1189.35 (11) Å^3^	

### Data collection

**Table:**

Bruker APEXII area-detector diffractometer	4208 independent reflections
Radiation source: fine-focus sealed tube	3790 reflections with *I* > 2σ(*I*)
graphite	*R*_int_ = 0.016
φ and ω scan	θ_max_ = 25.2°, θ_min_ = 1.6°
Absorption correction: multi-scan (*SADABS*; Sheldrick, 1996)	*h* = −10→8
*T*_min_ = 0.284, *T*_max_ = 0.378	*k* = −9→12
6174 measured reflections	*l* = −14→15

### Refinement

**Table:**

Refinement on *F*^2^	Primary atom site location: structure-invariant direct methods
Least-squares matrix: full	Secondary atom site location: difference Fourier map
*R*[*F*^2^ > 2σ(*F*^2^)] = 0.022	Hydrogen site location: inferred from neighbouring sites
*wR*(*F*^2^) = 0.054	H atoms treated by a mixture of independent and constrained refinement
*S* = 1.02	*w* = 1/[σ^2^(*F*_o_^2^) + (0.0292*P*)^2^ + 0.3497*P*] where *P* = (*F*_o_^2^ + 2*F*_c_^2^)/3
4208 reflections	(Δ/σ)_max_ = 0.002
397 parameters	Δρ_max_ = 0.79 e Å^−^^3^
17 restraints	Δρ_min_ = −0.81 e Å^−^^3^

### Special details

**Table:**

Geometry. All e.s.d.'s (except the e.s.d. in the dihedral angle between two l.s. planes) are estimated using the full covariance matrix. The cell e.s.d.'s are taken into account individually in the estimation of e.s.d.'s in distances, angles and torsion angles; correlations between e.s.d.'s in cell parameters are only used when they are defined by crystal symmetry. An approximate (isotropic) treatment of cell e.s.d.'s is used for estimating e.s.d.'s involving l.s. planes.
Refinement. Refinement of *F*^2^ against ALL reflections. The weighted *R*-factor *wR* and goodness of fit *S* are based on *F*^2^, conventional *R*-factors *R* are based on *F*, with *F* set to zero for negative *F*^2^. The threshold expression of *F*^2^ > σ(*F*^2^) is used only for calculating *R*-factors(gt) *etc*. and is not relevant to the choice of reflections for refinement. *R*-factors based on *F*^2^ are statistically about twice as large as those based on *F*, and *R*- factors based on ALL data will be even larger.

### Fractional atomic coordinates and isotropic or equivalent isotropic displacement parameters (Å^2^)

**Table:**

	*x*	*y*	*z*	*U*_iso_*/*U*_eq_	
Gd1	0.90952 (2)	0.549083 (19)	0.364770 (14)	0.01526 (7)	
Gd2	0.37043 (2)	0.410578 (19)	0.108204 (14)	0.01441 (7)	
Co1	0.35411 (7)	0.78823 (6)	0.27426 (4)	0.01829 (13)	
S1	0.92233 (13)	0.22231 (10)	0.40357 (8)	0.0196 (2)	
S2	0.77447 (12)	0.42234 (10)	0.09511 (7)	0.0157 (2)	
C1	0.7564 (5)	0.3980 (4)	0.5774 (3)	0.0152 (9)	
C2	0.5906 (5)	0.3349 (4)	0.5377 (3)	0.0175 (9)	
C3	0.3614 (5)	0.1987 (4)	0.5498 (3)	0.0224 (10)	
H3	0.2790	0.1358	0.5738	0.027*	
C4	0.4925 (5)	0.3440 (4)	0.4526 (3)	0.0176 (9)	
C5	0.5166 (5)	0.4170 (4)	0.3574 (3)	0.0179 (9)	
C6	0.5196 (5)	0.6976 (4)	0.1215 (3)	0.0183 (9)	
C7	0.5717 (5)	0.8417 (4)	0.1198 (3)	0.0198 (9)	
C8	0.5810 (6)	1.0356 (4)	0.1824 (3)	0.0262 (11)	
H8	0.5656	1.1038	0.2221	0.031*	
C9	0.6687 (5)	0.9312 (4)	0.0646 (3)	0.0212 (10)	
C10	0.7527 (5)	0.9224 (4)	−0.0265 (3)	0.0253 (10)	
N1	0.3480 (4)	0.2571 (4)	0.4626 (3)	0.0219 (8)	
N2	0.5066 (4)	0.2422 (3)	0.5971 (3)	0.0188 (8)	
N3	0.6720 (5)	1.0523 (3)	0.1057 (3)	0.0251 (9)	
N4	0.5169 (4)	0.9092 (3)	0.1936 (3)	0.0209 (8)	
O1	1.0507 (4)	0.2115 (3)	0.3471 (2)	0.0307 (8)	
O2	0.9891 (4)	0.2677 (3)	0.5141 (2)	0.0260 (7)	
O3	0.7976 (4)	0.0979 (3)	0.3993 (3)	0.0429 (10)	
O4	0.8622 (4)	0.3220 (3)	0.3559 (2)	0.0236 (7)	
O5	0.8508 (3)	0.4831 (3)	0.5314 (2)	0.0191 (6)	
O6	0.8087 (3)	0.3626 (3)	0.6615 (2)	0.0195 (6)	
O7	0.6446 (3)	0.5035 (3)	0.3536 (2)	0.0227 (7)	
O8	0.4026 (4)	0.3819 (3)	0.2864 (2)	0.0291 (8)	
O9	0.4237 (4)	0.6481 (3)	0.1835 (2)	0.0216 (7)	
O10	0.5663 (3)	0.6222 (3)	0.0648 (2)	0.0201 (7)	
O11	0.7322 (4)	0.8084 (3)	−0.0689 (3)	0.0373 (9)	
O12	0.8336 (4)	1.0256 (3)	−0.0559 (3)	0.0371 (9)	
O13	0.7800 (4)	0.5049 (3)	0.0081 (2)	0.0261 (7)	
O14	0.8239 (4)	0.3108 (3)	0.0655 (2)	0.0302 (8)	
O15	0.8852 (4)	0.5027 (3)	0.1839 (2)	0.0315 (8)	
O16	0.6158 (4)	0.3748 (3)	0.1261 (2)	0.0296 (8)	
H1	0.260 (4)	0.251 (5)	0.426 (3)	0.044*	
H2	0.725 (5)	1.128 (3)	0.087 (4)	0.044*	
O1W	0.8720 (5)	0.7325 (3)	0.2745 (3)	0.0411 (9)	
H1W	0.931 (6)	0.807 (3)	0.275 (4)	0.062*	
H2W	0.843 (7)	0.703 (5)	0.216 (2)	0.062*	
O2W	0.1300 (4)	0.3676 (4)	0.1756 (3)	0.0301 (8)	
H3W	0.115 (5)	0.331 (5)	0.227 (2)	0.045*	
H4W	0.046 (4)	0.359 (5)	0.143 (3)	0.045*	
O3W	0.1637 (4)	0.7554 (3)	0.1538 (2)	0.0305 (8)	
H5W	0.166 (7)	0.811 (3)	0.113 (3)	0.046*	
H6W	0.131 (6)	0.686 (3)	0.114 (3)	0.046*	
O4W	0.2924 (5)	0.9293 (4)	0.3611 (3)	0.0402 (9)	
H7W	0.267 (6)	0.916 (6)	0.419 (2)	0.060*	
H8W	0.221 (5)	0.949 (6)	0.330 (4)	0.060*	
O5W	0.0564 (5)	−0.0166 (4)	0.2481 (3)	0.0440 (10)	
H9W	0.079 (7)	−0.006 (5)	0.187 (2)	0.066*	
H10W	0.028 (7)	0.046 (4)	0.271 (4)	0.066*	

### Atomic displacement parameters (Å^2^)

**Table:**

	*U*^11^	*U*^22^	*U*^33^	*U*^12^	*U*^13^	*U*^23^
Gd1	0.01463 (12)	0.01957 (12)	0.01160 (11)	0.00505 (9)	0.00226 (8)	0.00460 (8)
Gd2	0.01453 (12)	0.01764 (12)	0.01186 (11)	0.00550 (9)	0.00300 (8)	0.00492 (8)
Co1	0.0212 (3)	0.0206 (3)	0.0141 (3)	0.0069 (2)	0.0050 (2)	0.0054 (2)
S1	0.0214 (6)	0.0180 (5)	0.0181 (5)	0.0053 (4)	0.0011 (4)	0.0022 (4)
S2	0.0164 (5)	0.0211 (5)	0.0111 (5)	0.0077 (4)	0.0024 (4)	0.0041 (4)
C1	0.020 (2)	0.017 (2)	0.0083 (19)	0.0053 (18)	0.0044 (17)	0.0013 (16)
C2	0.022 (2)	0.021 (2)	0.0102 (19)	0.0082 (18)	0.0032 (17)	0.0004 (17)
C3	0.021 (2)	0.024 (2)	0.020 (2)	0.0035 (19)	0.0038 (19)	0.0055 (18)
C4	0.018 (2)	0.021 (2)	0.013 (2)	0.0056 (18)	0.0011 (17)	0.0031 (17)
C5	0.017 (2)	0.029 (2)	0.010 (2)	0.0095 (19)	0.0058 (17)	0.0057 (17)
C6	0.018 (2)	0.020 (2)	0.015 (2)	0.0039 (18)	−0.0011 (18)	0.0041 (18)
C7	0.023 (2)	0.018 (2)	0.018 (2)	0.0057 (18)	0.0050 (18)	0.0017 (17)
C8	0.038 (3)	0.017 (2)	0.025 (2)	0.009 (2)	0.012 (2)	0.0031 (19)
C9	0.026 (3)	0.014 (2)	0.021 (2)	0.0034 (18)	0.0045 (19)	0.0031 (18)
C10	0.027 (3)	0.024 (3)	0.023 (2)	0.003 (2)	0.010 (2)	0.005 (2)
N1	0.016 (2)	0.031 (2)	0.0177 (19)	0.0067 (17)	0.0005 (15)	0.0044 (16)
N2	0.020 (2)	0.0204 (19)	0.0142 (17)	0.0039 (15)	0.0035 (15)	0.0047 (14)
N3	0.035 (2)	0.0154 (19)	0.025 (2)	0.0043 (17)	0.0127 (18)	0.0081 (16)
N4	0.024 (2)	0.0188 (19)	0.0185 (18)	0.0050 (16)	0.0045 (16)	0.0011 (15)
O1	0.033 (2)	0.046 (2)	0.0184 (16)	0.0199 (16)	0.0057 (14)	0.0000 (15)
O2	0.037 (2)	0.0269 (17)	0.0154 (15)	0.0120 (15)	0.0022 (14)	0.0038 (13)
O3	0.031 (2)	0.0262 (19)	0.057 (2)	−0.0065 (15)	−0.0102 (18)	0.0148 (17)
O4	0.0266 (18)	0.0222 (16)	0.0229 (16)	0.0107 (14)	−0.0012 (14)	0.0020 (13)
O5	0.0160 (16)	0.0252 (16)	0.0149 (14)	0.0041 (13)	0.0030 (12)	0.0064 (12)
O6	0.0167 (16)	0.0296 (17)	0.0125 (14)	0.0070 (13)	0.0029 (12)	0.0074 (12)
O7	0.0161 (16)	0.0295 (17)	0.0225 (16)	0.0057 (13)	0.0029 (13)	0.0122 (13)
O8	0.0191 (17)	0.048 (2)	0.0167 (16)	0.0052 (15)	0.0010 (13)	0.0133 (15)
O9	0.0276 (18)	0.0182 (15)	0.0193 (15)	0.0049 (13)	0.0105 (13)	0.0067 (12)
O10	0.0246 (17)	0.0149 (15)	0.0209 (15)	0.0048 (13)	0.0084 (13)	0.0036 (12)
O11	0.053 (2)	0.0187 (18)	0.037 (2)	0.0021 (16)	0.0258 (18)	0.0011 (15)
O12	0.050 (2)	0.0236 (18)	0.039 (2)	0.0061 (16)	0.0248 (18)	0.0103 (15)
O13	0.0230 (18)	0.042 (2)	0.0184 (16)	0.0142 (15)	0.0053 (13)	0.0170 (14)
O14	0.039 (2)	0.0200 (17)	0.0346 (18)	0.0095 (15)	0.0187 (16)	0.0038 (14)
O15	0.035 (2)	0.0346 (19)	0.0158 (16)	0.0001 (15)	0.0002 (14)	−0.0013 (14)
O16	0.0236 (18)	0.045 (2)	0.0275 (17)	0.0164 (15)	0.0122 (14)	0.0208 (15)
O1W	0.053 (3)	0.029 (2)	0.035 (2)	0.0081 (18)	−0.0066 (19)	0.0089 (16)
O2W	0.0199 (18)	0.049 (2)	0.0271 (18)	0.0154 (16)	0.0092 (14)	0.0182 (16)
O3W	0.035 (2)	0.0295 (18)	0.0251 (17)	0.0100 (16)	−0.0016 (15)	0.0045 (14)
O4W	0.055 (3)	0.041 (2)	0.032 (2)	0.0265 (19)	0.0098 (18)	0.0015 (18)
O5W	0.063 (3)	0.040 (2)	0.043 (2)	0.028 (2)	0.026 (2)	0.0114 (18)

### Geometric parameters (Å, °)

**Table:**

Gd1—O7	2.283 (3)	C3—N2	1.314 (5)
Gd1—O2^i^	2.319 (3)	C3—N1	1.336 (5)
Gd1—O4	2.337 (3)	C3—H3	0.9300
Gd1—O15	2.343 (3)	C4—N1	1.370 (5)
Gd1—O5	2.375 (3)	C4—C5	1.498 (5)
Gd1—O1W	2.447 (3)	C5—O7	1.242 (5)
Gd1—O6^i^	2.498 (3)	C5—O8	1.249 (5)
Gd1—O5^i^	2.562 (3)	C6—O9	1.263 (5)
Gd1—C1^i^	2.905 (4)	C6—O10	1.268 (5)
Gd1—Gd1^i^	4.0465 (4)	C6—C7	1.471 (6)
Gd2—O11^ii^	2.244 (3)	C7—C9	1.373 (6)
Gd2—O13^ii^	2.338 (3)	C7—N4	1.399 (5)
Gd2—O8	2.348 (3)	C8—N4	1.320 (5)
Gd2—O2W	2.361 (3)	C8—N3	1.347 (6)
Gd2—O16	2.373 (3)	C8—H8	0.9300
Gd2—O10^ii^	2.414 (3)	C9—N3	1.372 (5)
Gd2—O10	2.535 (3)	C9—C10	1.493 (6)
Gd2—O9	2.561 (3)	C10—O12	1.226 (5)
Gd2—C6	2.934 (4)	C10—O11	1.265 (5)
Gd2—Gd2^ii^	4.0349 (4)	N1—H1	0.87 (4)
Co1—N4	2.058 (4)	N2—Co1^iii^	2.086 (3)
Co1—N2^iii^	2.086 (3)	N3—H2	0.87 (3)
Co1—O6^iii^	2.096 (3)	O2—Gd1^i^	2.319 (3)
Co1—O4W	2.097 (4)	O5—Gd1^i^	2.562 (3)
Co1—O3W	2.122 (3)	O6—Co1^iii^	2.096 (3)
Co1—O9	2.157 (3)	O6—Gd1^i^	2.498 (3)
S1—O3	1.443 (3)	O10—Gd2^ii^	2.414 (3)
S1—O1	1.478 (3)	O11—Gd2^ii^	2.244 (3)
S1—O2	1.483 (3)	O13—Gd2^ii^	2.338 (3)
S1—O4	1.486 (3)	O1W—H1W	0.81 (4)
S2—O14	1.459 (3)	O1W—H2W	0.789 (19)
S2—O15	1.469 (3)	O2W—H3W	0.80 (3)
S2—O13	1.470 (3)	O2W—H4W	0.81 (4)
S2—O16	1.476 (3)	O3W—H5W	0.82 (3)
C1—O5	1.264 (5)	O3W—H6W	0.83 (3)
C1—O6	1.269 (5)	O4W—H7W	0.82 (3)
C1—C2	1.458 (6)	O4W—H8W	0.82 (5)
C1—Gd1^i^	2.905 (4)	O5W—H9W	0.85 (4)
C2—C4	1.369 (6)	O5W—H10W	0.85 (5)
C2—N2	1.377 (5)		
			
O7—Gd1—O2^i^	103.56 (11)	O4W—Co1—O3W	93.45 (14)
O7—Gd1—O4	87.61 (10)	N4—Co1—O9	78.00 (12)
O2^i^—Gd1—O4	139.04 (10)	N2^iii^—Co1—O9	87.71 (13)
O7—Gd1—O15	90.06 (11)	O6^iii^—Co1—O9	91.82 (11)
O2^i^—Gd1—O15	137.61 (11)	O4W—Co1—O9	178.18 (14)
O4—Gd1—O15	80.42 (11)	O3W—Co1—O9	87.04 (13)
O7—Gd1—O5	76.05 (10)	O3—S1—O1	112.2 (2)
O2^i^—Gd1—O5	71.43 (10)	O3—S1—O2	108.8 (2)
O4—Gd1—O5	73.46 (10)	O1—S1—O2	107.78 (19)
O15—Gd1—O5	150.71 (11)	O3—S1—O4	110.53 (19)
O7—Gd1—O1W	77.94 (12)	O1—S1—O4	107.51 (18)
O2^i^—Gd1—O1W	74.57 (11)	O2—S1—O4	110.02 (17)
O4—Gd1—O1W	146.18 (11)	O14—S2—O15	108.6 (2)
O15—Gd1—O1W	69.33 (12)	O14—S2—O13	109.56 (18)
O5—Gd1—O1W	130.26 (12)	O15—S2—O13	107.94 (19)
O7—Gd1—O6^i^	164.37 (9)	O14—S2—O16	110.07 (19)
O2^i^—Gd1—O6^i^	76.75 (10)	O15—S2—O16	109.10 (19)
O4—Gd1—O6^i^	102.43 (10)	O13—S2—O16	111.47 (17)
O15—Gd1—O6^i^	80.01 (10)	O5—C1—O6	118.6 (4)
O5—Gd1—O6^i^	118.09 (9)	O5—C1—C2	123.7 (3)
O1W—Gd1—O6^i^	87.23 (12)	O6—C1—C2	117.7 (3)
O7—Gd1—O5^i^	144.56 (9)	O5—C1—Gd1^i^	61.8 (2)
O2^i^—Gd1—O5^i^	75.17 (10)	O6—C1—Gd1^i^	58.9 (2)
O4—Gd1—O5^i^	73.51 (10)	C2—C1—Gd1^i^	163.6 (3)
O15—Gd1—O5^i^	115.09 (11)	C4—C2—N2	109.0 (4)
O5—Gd1—O5^i^	69.99 (11)	C4—C2—C1	135.5 (4)
O1W—Gd1—O5^i^	132.85 (11)	N2—C2—C1	115.5 (3)
O6^i^—Gd1—O5^i^	50.97 (9)	N2—C3—N1	110.9 (4)
O7—Gd1—C1^i^	168.66 (10)	N2—C3—H3	124.5
O2^i^—Gd1—C1^i^	70.40 (11)	N1—C3—H3	124.5
O4—Gd1—C1^i^	91.09 (11)	C2—C4—N1	105.5 (3)
O15—Gd1—C1^i^	100.83 (11)	C2—C4—C5	133.9 (4)
O5—Gd1—C1^i^	92.78 (10)	N1—C4—C5	120.4 (4)
O1W—Gd1—C1^i^	108.73 (12)	O7—C5—O8	125.4 (4)
O6^i^—Gd1—C1^i^	25.79 (9)	O7—C5—C4	119.5 (4)
O5^i^—Gd1—C1^i^	25.77 (10)	O8—C5—C4	115.1 (4)
O7—Gd1—Gd1^i^	111.99 (7)	O9—C6—O10	119.1 (4)
O2^i^—Gd1—Gd1^i^	69.58 (7)	O9—C6—C7	117.3 (4)
O4—Gd1—Gd1^i^	69.70 (7)	O10—C6—C7	123.6 (4)
O15—Gd1—Gd1^i^	141.31 (9)	O9—C6—Gd2	60.5 (2)
O5—Gd1—Gd1^i^	36.51 (7)	O10—C6—Gd2	59.4 (2)
O1W—Gd1—Gd1^i^	144.09 (9)	C7—C6—Gd2	171.4 (3)
O6^i^—Gd1—Gd1^i^	82.97 (6)	C9—C7—N4	109.2 (4)
O5^i^—Gd1—Gd1^i^	33.48 (6)	C9—C7—C6	135.5 (4)
C1^i^—Gd1—Gd1^i^	57.20 (7)	N4—C7—C6	115.3 (4)
O11^ii^—Gd2—O13^ii^	104.05 (12)	N4—C8—N3	110.6 (4)
O11^ii^—Gd2—O8	90.51 (12)	N4—C8—H8	124.7
O13^ii^—Gd2—O8	138.78 (11)	N3—C8—H8	124.7
O11^ii^—Gd2—O2W	79.70 (13)	N3—C9—C7	105.2 (4)
O13^ii^—Gd2—O2W	75.63 (11)	N3—C9—C10	119.4 (4)
O8—Gd2—O2W	69.29 (11)	C7—C9—C10	135.2 (4)
O11^ii^—Gd2—O16	85.00 (13)	O12—C10—O11	125.0 (4)
O13^ii^—Gd2—O16	139.36 (10)	O12—C10—C9	117.9 (4)
O8—Gd2—O16	79.29 (11)	O11—C10—C9	117.1 (4)
O2W—Gd2—O16	144.68 (10)	C3—N1—C4	108.3 (3)
O11^ii^—Gd2—O10^ii^	76.41 (10)	C3—N1—H1	125 (4)
O13^ii^—Gd2—O10^ii^	71.52 (10)	C4—N1—H1	126 (4)
O8—Gd2—O10^ii^	149.64 (11)	C3—N2—C2	106.3 (3)
O2W—Gd2—O10^ii^	132.68 (11)	C3—N2—Co1^iii^	140.8 (3)
O16—Gd2—O10^ii^	72.45 (10)	C2—N2—Co1^iii^	112.8 (3)
O11^ii^—Gd2—O10	145.26 (11)	C8—N3—C9	109.0 (4)
O13^ii^—Gd2—O10	76.33 (10)	C8—N3—H2	125 (4)
O8—Gd2—O10	112.31 (10)	C9—N3—H2	126 (4)
O2W—Gd2—O10	131.93 (11)	C8—N4—C7	106.0 (4)
O16—Gd2—O10	74.61 (10)	C8—N4—Co1	139.7 (3)
O10^ii^—Gd2—O10	70.80 (11)	C7—N4—Co1	114.0 (3)
O11^ii^—Gd2—O9	163.85 (11)	S1—O2—Gd1^i^	144.35 (18)
O13^ii^—Gd2—O9	74.78 (10)	S1—O4—Gd1	141.43 (18)
O8—Gd2—O9	80.86 (10)	C1—O5—Gd1	143.6 (3)
O2W—Gd2—O9	84.47 (11)	C1—O5—Gd1^i^	92.4 (2)
O16—Gd2—O9	106.54 (11)	Gd1—O5—Gd1^i^	110.01 (11)
O10^ii^—Gd2—O9	117.44 (9)	C1—O6—Co1^iii^	115.4 (3)
O10—Gd2—O9	50.71 (9)	C1—O6—Gd1^i^	95.3 (2)
O11^ii^—Gd2—C6	169.70 (11)	Co1^iii^—O6—Gd1^i^	143.17 (13)
O13^ii^—Gd2—C6	71.59 (11)	C5—O7—Gd1	145.3 (3)
O8—Gd2—C6	98.84 (11)	C5—O8—Gd2	134.4 (3)
O2W—Gd2—C6	107.59 (12)	C6—O9—Co1	115.0 (3)
O16—Gd2—C6	92.46 (12)	C6—O9—Gd2	94.1 (2)
O10^ii^—Gd2—C6	93.30 (11)	Co1—O9—Gd2	149.36 (13)
O10—Gd2—C6	25.50 (10)	C6—O10—Gd2^ii^	142.3 (3)
O9—Gd2—C6	25.43 (10)	C6—O10—Gd2	95.1 (2)
O11^ii^—Gd2—Gd2^ii^	112.07 (8)	Gd2^ii^—O10—Gd2	109.20 (11)
O13^ii^—Gd2—Gd2^ii^	70.24 (7)	C10—O11—Gd2^ii^	159.9 (3)
O8—Gd2—Gd2^ii^	139.01 (7)	S2—O13—Gd2^ii^	144.17 (19)
O2W—Gd2—Gd2^ii^	145.64 (8)	S2—O15—Gd1	141.6 (2)
O16—Gd2—Gd2^ii^	69.68 (7)	S2—O16—Gd2	142.69 (18)
O10^ii^—Gd2—Gd2^ii^	36.40 (6)	Gd1—O1W—H1W	130 (4)
O10—Gd2—Gd2^ii^	34.41 (6)	Gd1—O1W—H2W	104 (4)
O9—Gd2—Gd2^ii^	82.98 (6)	H1W—O1W—H2W	108 (3)
C6—Gd2—Gd2^ii^	57.82 (8)	Gd2—O2W—H3W	122 (4)
N4—Co1—N2^iii^	102.68 (14)	Gd2—O2W—H4W	127 (3)
N4—Co1—O6^iii^	169.67 (13)	H3W—O2W—H4W	108 (3)
N2^iii^—Co1—O6^iii^	78.43 (12)	Co1—O3W—H5W	120 (4)
N4—Co1—O4W	100.20 (15)	Co1—O3W—H6W	121 (4)
N2^iii^—Co1—O4W	92.40 (14)	H5W—O3W—H6W	102 (3)
O6^iii^—Co1—O4W	89.98 (14)	Co1—O4W—H7W	121 (4)
N4—Co1—O3W	94.51 (14)	Co1—O4W—H8W	113 (4)
N2^iii^—Co1—O3W	160.56 (13)	H7W—O4W—H8W	104 (3)
O6^iii^—Co1—O3W	83.04 (12)	H9W—O5W—H10W	110 (3)

Symmetry codes: (i) −*x*+2, −*y*+1, −*z*+1; (ii) −*x*+1, −*y*+1, −*z*; (iii) −*x*+1, −*y*+1, −*z*+1.

### Hydrogen-bond geometry (Å, °)

**Table:**

*D*—H···*A*	*D*—H	H···*A*	*D*···*A*	*D*—H···*A*
N1—H1···O1^iv^	0.87 (4)	1.96 (4)	2.820 (5)	172 (5)
O1W—H1W···O5W^v^	0.81 (4)	1.95 (3)	2.745 (5)	169 (6)
N3—H2···O14^vi^	0.87 (3)	1.93 (3)	2.787 (4)	169 (4)
O2W—H3W···O1^iv^	0.80 (3)	2.09 (4)	2.878 (5)	171 (5)
O2W—H4W···O14^iv^	0.81 (4)	2.04 (4)	2.842 (5)	172 (5)
O2W—H4W···O15^iv^	0.81 (4)	2.52 (4)	3.035 (5)	123 (4)
O3W—H5W···O12^vii^	0.82 (3)	1.95 (4)	2.734 (4)	162 (5)
O3W—H6W···O14^ii^	0.83 (3)	2.41 (4)	2.919 (4)	120 (3)
O4W—H7W···O3^iii^	0.82 (3)	2.49 (3)	3.306 (6)	174 (6)
O4W—H8W···O5W^vi^	0.82 (5)	1.89 (5)	2.700 (6)	175 (6)
O5W—H9W···O12^ii^	0.85 (4)	1.99 (4)	2.797 (6)	161 (5)
O5W—H10W···O1^iv^	0.85 (5)	1.93 (5)	2.728 (5)	157 (6)
C3—H3···O3^viii^	0.93	2.44	3.193 (5)	138.

Symmetry codes: (iv) *x*−1, *y*, *z*; (v) *x*+1, *y*+1, *z*; (vi) *x*, *y*+1, *z*; (vii) −*x*+1, −*y*+2, −*z*; (ii) −*x*+1, −*y*+1, −*z*; (iii) −*x*+1, −*y*+1, −*z*+1; (viii) −*x*+1, −*y*, −*z*+1.
